# Supplementary figures and images for: Comparison of orbital structures between age-related distance esotropia and acute acquired concomitant esotropia
Source: PLoS One. 2025 May 27;20(5):e0324078. doi: 10.1371/journal.pone.0324078 (PMC12111637; doi:10.1371/journal.pone.0324078)

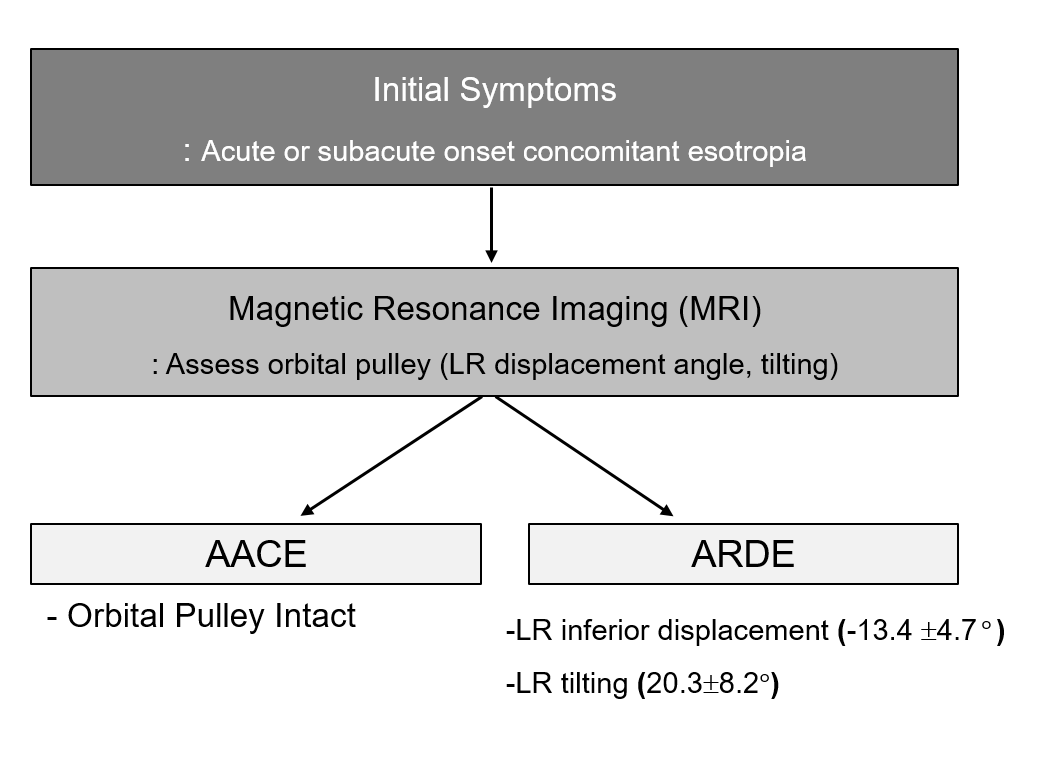

Supplement: S1 Fig — (TIF) [file pone.0324078.s001.tif]
